# Zika Virus Induces Tumor Necrosis Factor-Related Apoptosis Inducing Ligand (TRAIL)-Mediated Apoptosis in Human Neural Progenitor Cells

**DOI:** 10.3390/cells9112487

**Published:** 2020-11-16

**Authors:** Jae Kyung Lee, Ji-Ae Kim, Soo-Jin Oh, Eun-Woo Lee, Ok Sarah Shin

**Affiliations:** 1 BK21 Graduate Program, Department of Biomedical Sciences, Korea University College of Medicine, Seoul 08308, Korea; jae.lee0321@gmail.com (J.K.L.); kja0910kr@gmail.com (J.-A.K.); sjooooh@gmail.com (S.-J.O.); 2 Metabolic Regulation Research Center, Korea Research Institute of Bioscience and Biotechnology (KRIBB), Daejeon 34141, Korea

**Keywords:** Zika virus, apoptosis, TRAIL, FADD

## Abstract

Zika virus (ZIKV) remains as a public health threat due to the congenital birth defects the virus causes following infection of pregnant women. Congenital microcephaly is among the neurodevelopmental disorders the virus can cause in newborns, and this defect has been associated with ZIKV-mediated cytopathic effects in human neural progenitor cells (hNPCs). In this study, we investigated the cellular changes that occur in hNPCs in response to ZIKV (African and Asian lineages)-induced cytopathic effects. Transmission electron microscopy showed the progress of cell death as well as the formation of numerous vacuoles in the cytoplasm of ZIKV-infected hNPCs. Infection with both African and Asian lineages of ZIKV induced apoptosis, as demonstrated by the increased activation of caspase 3/7, 8, and 9. Increased levels of proinflammatory cytokines and chemokines (IL-6, IL-8, IL-1β) were also detected in ZIKV-infected hNPCs, while z-VAD-fmk-induced inhibition of cell death suppressed ZIKV-mediated cytokine production in a dose-dependent manner. ZIKV-infected hNPCs also displayed significantly elevated gene expression levels of the pro-apoptotic Bcl2-mediated family, in particular, tumor necrosis factor-related apoptosis-inducing ligand (TRAIL). Furthermore, TRAIL signaling led to augmented ZIKV-mediated cell death and the knockdown of TRAIL-mediated signaling adaptor, FADD, resulted in enhanced ZIKV replication. In conclusion, our findings provide cellular insights into the cytopathic effects induced by ZIKV infection of hNPCs.

## 1. Introduction

Zika virus (ZIKV) is a positive sense RNA virus belonging to the *Flaviviridae* family, and was first isolated from a febrile rhesus monkey in the Zika forest of Uganda in 1947 [1]. Although mosquito-mediated transmission is the primary route responsible for the epidemic spread, ZIKV can also be transmitted to humans by non-vector-mediated mechanisms, including sexual interactions, blood transfusion, and mother-to-fetus transmission during all trimesters of pregnancy [2,3,4]. After the onset of the 2015 epidemic in South America, ZIKV was identified as a causative agent of severe birth defects, such as microcephaly and cerebral calcifications, following in utero exposure to the virus [5]. At present, ZIKV continues to pose a major threat to public health due to congenital abnormalities associated with ZIKV infection during pregnancy. Currently, there is no licensed vaccine or specific antiviral therapy available to prevent or treat ZIKV infections.

The following defects during neurogenesis have been shown to be responsible for congenital microcephaly: depletion of NPCs due to apoptosis and/or premature differentiation, inhibition of NPC proliferation, or apoptosis of newly generated neurons. The cellular tropism of infection of ZIKV is evident from the ability of the virus to replicate and induce cell death in neural progenitor cells and brain organoids, and this cell death mechanism plays an important role during the pathogenesis of ZIKV-associated diseases [6,7,8,9,10,11,12,13]. ZIKV reduces NPC proliferation, induces their premature differentiation, and activates apoptosis of NPCs and immature neurons [14]. In terms of cell death pathways activated by the inflammatory response, pyroptosis, necrosis, and necroptosis have also been studied in the context of ZIKV infection and microcephaly [15,16,17]. Nevertheless, a question remains as to the detailed mechanisms through which ZIKV causes cytotoxic effects during neurogenesis.

Type I and III interferons (IFNs) are well-known signaling molecules during immune responses responsible for controlling viral infections, and activation of IFN signaling results in the production of IFN-stimulated genes (ISGs), including TRAIL [18]. TRAIL is a member of the tumor necrosis factor (TNF) family of ligands of death receptors that are able to kill target cells as part of the host immune response. TRAIL is expressed on different cells of the immune system and selectively induces apoptosis of a variety of tumor cells and virus-infected cells, but not most normal cells. Previous reports have highlighted TRAIL as a host-derived signaling mediator that is implicated in viral infections, during which TRAIL can either participate in pro- or antiviral responses. TRAIL can induce virus-infected cells to undergo cell death, but the mediator can also induce uninfected cells to undergo apoptosis and necrosis [19,20,21,22]. FAS-associating protein with death domain (FADD) is an adaptor protein that is recruited upon the activation of TRAIL receptors, and the interaction between death receptors and adaptor proteins has also been reported to trigger the initiation of the caspase activation cascade [23]. Although different types of cell death mechanisms have been studied following ZIKV infection, the specific role of TRAIL has not been investigated in the context of ZIKV-induced cell death pathways.

Given that both apoptosis and necroptosis have been implicated in cases of ZIKV-induced microcephaly, we examined ZIKV-induced neuronal cell death and modulation of cell growth or apoptosis signaling in the presence of caspase or necroptosis inhibitors. Our data demonstrate ZIKV induces tumor necrosis factor-related apoptosis-inducing ligand (TRAIL)-mediated apoptosis in hNPCs, and FADD knockdown can suppress cell death induced by ZIKV to enhance ZIKV replication.

## 2. Materials and Methods

### 2.1. Cells, Viruses, and Reagents

A549 and African green monkey kidney epithelial (Vero) cells obtained from American Type Culture Collection (ATCC; Manassas, VA, USA) were used for this study. A549 cells were cultured at 37 °C in RPMI 1640 medium (Corning Mediatech, Corning, NY, USA) supplemented with 10% fetal bovine serum (FBS; Corning Mediatech) and 1% antibiotics. Vero cells were cultured at 37 °C in Dulbecco’s modified Eagle’s medium (DMEM; Corning Mediatech) supplemented with 10% FBS and 1% antibiotics. Human neural progenitor cells (hNPCs) were described previously [24,25,26]. Briefly, human embryonic stem cells (hESCs) were grown under standard culture conditions with DMEM/F12 supplemented with 2% B27, 100 ng/mL fibroblast growth factor, 100 ng/mL epidermal growth factor, and 5 μg/mL heparin. Partially differentiated hESCs were dissociated with accutase and plated on geltrex-coated plates. Homogenous populations of NPCs were obtained after three continuous passages. Matrigel was purchased from BD Biosciences, San Jose, CA, USA and other reagents were purchased from Invitrogen, Carlsbad, CA, USA.

ZIKV MR766 (African lineage) and PRVABC59 (Asian lineage) strains were purchased from ATCC and propagated in Vero cells. Viral titers were determined using a standard plaque assay as described previously [27]. Viral stocks were aliquoted and stored at −80 °C until use. For the measurement of tissue culture infective dose (TCID50), supernatants from ZIKV-infected hNPCs were collected at 24, 48, and 72 hpi. TCID50 was determined using the Spearman–Kärber method and expressed as TCID50/mL.

### 2.2. ZIKV Infection and Drug Treatment

The following drugs purchased from Sigma (St Louis, MO, USA) were used to study ZIKV-induced cell death: TNF-α (25 ng/mL), cycloheximide (CHX, 5 µg/mL), z-VAD-fmk (10 µM), Necrostatin (Nec-1, 30 µg/mL), GSK872 (5 µM), and NSA (5 µM). TRAIL was purchased from Peprotech (Cranbury, NJ, USA). Cells were treated with drug prior to infection overnight or 4 h before the infection. During infection, drug was removed and was introduced immediately after infection until sampling. 

### 2.3. Transmission Electron Microscopy (TEM)

Mock- or ZIKV-infected hNPCs were pelleted and washed twice with PBS. Fixation was performed with PBS pH 7.4 containing 2.5% glutaraldehyde and 2% paraformaldehyde for 30 min at 4 °C. The pellets were rinsed twice with cold PBS, post-fixed in 1% osmium tetroxide, dehydrated in ethanol series, incubated twice with propylene oxide for 20 min, and embedded in Epon mixture. Ultrathin sections of 70 nm were obtained using a Reichert-Jung Ultracut E ultramicrotome (Leica, Wetzlar), mounted on copper grids, and counterstained with uranyl acetate and lead citrate. The specimens were observed with a Hitachi H-7600 electron microscope (Hitachi, Japan) at 80 kV acceleration voltage.

### 2.4. Cell Viability and Cytotoxicity Assay

Cells were seeded in 96-well plates overnight. Samples in triplicate were then infected with ZIKV at indicated MOIs for various time points. Cell survival was monitored by CellTiter-Glo^®^ Luminescent Cell Viability Assay (Promega, Madison, WI, USA) as per the manufacturer’s instructions. Mock samples were set to 100% survival and all other samples were expressed relative to mock.

Cell death was determined by the extracellular release of lactate dehydrogenase (LDH) using a CytoTox96 non-radioactive cytotoxicity assay kit (Promega) according to the manufacturer’s instructions. LDH release was calculated as [extracellular LDH/(intracellular LDH + extracellular LDH) × 100].

### 2.5. Flow Cytometry

Cells were harvested and fixed with 95% ethanol containing 0.5% Tween-20 for 24 h before washing with PBS and staining with propidium iodide (PI, 50 µg/mL) and RNase (50 µg/mL) for 30 min. For Annexin V-FITC/PI assay, cells were stained using a FITC-conjugated Annexin V apoptosis detection kit (BD Bioscience, Franklin Lakes, NJ, USA) according to the manufacturer’s protocol. Stained cells were analyzed with BD LSR Fortessa™ X-20 Cell Analyzer (BD Bioscience, Piscataway, NJ, USA).

### 2.6. Caspase Activity Assay

The activity of caspases 3/7, 8, and 9 was measured with a Caspase-Glo assay kit, according to the manufacturer’s instructions (Promega, Madison, WI, USA). Briefly, 100 μL of Caspase-Glo reagent were added to each well, mixed with a plate shaker at 300–500 rpm, and incubated at room temperature for 2 h. The luminescence of each sample was measured in a Varioskan LUX multimode microplate reader (Thermo Fisher Scientific, Waltham, MA, USA).

### 2.7. Quantitative Real-Time PCR (qRT-PCR)

Total RNA (0.5 μg) was isolated with Trizol reagent (Invitrogen) and reverse transcribed to generate cDNA using an ImProm-II Reverse Transcription System (Promega, Madison, WI, USA) for 1 h at 42 °C. The resulting cDNA was used as a template for qRT-PCR quantification of ZIKV and host transcript levels using a Power SYBR Green PCR Master Mix (Thermo Fisher Scientific, Waltham, MA, USA). The primer sequences used in this study are listed in Table 1 [27]. Quantification was carried out on a QuantStudio 6 Flex Real-time PCR system (Thermo Fisher Scientific, Waltham, MA, USA) for cDNA amplification with Power SYBR^®^ Green Master Mix (Invitrogen) under the following conditions: 95 ℃ for 10 min, followed by 40 cycles of 95 ℃ for 30 s, and 60 ℃ for 1 min. Relative mRNA levels were determined using the comparative Ct method and normalized against β-actin mRNA.

### 2.8. Western Blotting

Cells were lysed at the specified time points using RIPA buffer (Sigma-Aldrich). Lysates were separated by sodium dodecyl sulfate polyacrylamide gel electrophoresis (SDS-PAGE) on 10–12% acrylamide gels. Proteins were transferred to polyvinylidene difluoride (PVDF) membranes and blocked with 5% (*w*/*v*) skim milk in Tris-buffered saline (0.2 M Tris, 1.36 M NaCl) supplemented with 0.1% (*v*/*v*) Tween-20 (TBS-Tw) for 1 h at 25 °C as described previously [26]. This was followed by an overnight incubation with primary antibodies (Cell Signaling Technologies, Danvers, MA, USA) at 4 °C. After three washes in TBS/Tween-20, the membranes were incubated with HRP-conjugated anti-rabbit or anti-mouse IgG secondary antibodies for 1 h at 25 °C. Membranes were washed with TBS/Tween-20, incubated with Western Lumi Pico solution (ECL solution kit; DoGen, Seoul, Korea). Signals were determined using a Fusion Solo Imaging System (Vilber Lourmat, Collégien, France). Band intensities were quantified by Fusion-Capt analysis software (Vilber Lourmat, Collégien, France).

### 2.9. siRNA Transfection

Cells were seeded in 6-well plates and cultured until 70% confluency on the day of transfection. Transient transfections with control scrambled RIG-I- or FADD-specific siRNAs (Bioneer, Daejeon, Korea) were performed with RNAiMAX^®^ transfection reagent (Invitrogen) according to the manufacturer’s protocol [28,29].

### 2.10. Enzyme-Linked Immunosorbent Assay (ELISA)

TNF-α, IL-6, IL-8, and IL-1β ELISA kits were purchased from R&D Systems (Minneapolis, MI, USA). The assay was performed according to the manufacturer’s instructions. Absorbance at 450 nm was measured using a microplate spectrophotometer.

### 2.11. Statistical Analysis

Quantitative data were expressed as means ± standard deviation (SD). Statistical analysis was performed using Graphpad Prism (Graphpad Software, La Jolla, CA, USA) by comparing controls to treated groups. Student’s *t* tests or one-way ANOVA tests were performed to compare individual treatments.

## 3. Results

### 3.1. Both African and Asian Lineage ZIKV Infection Results in the Activation of Apoptotic Signaling

**Figure 1:**
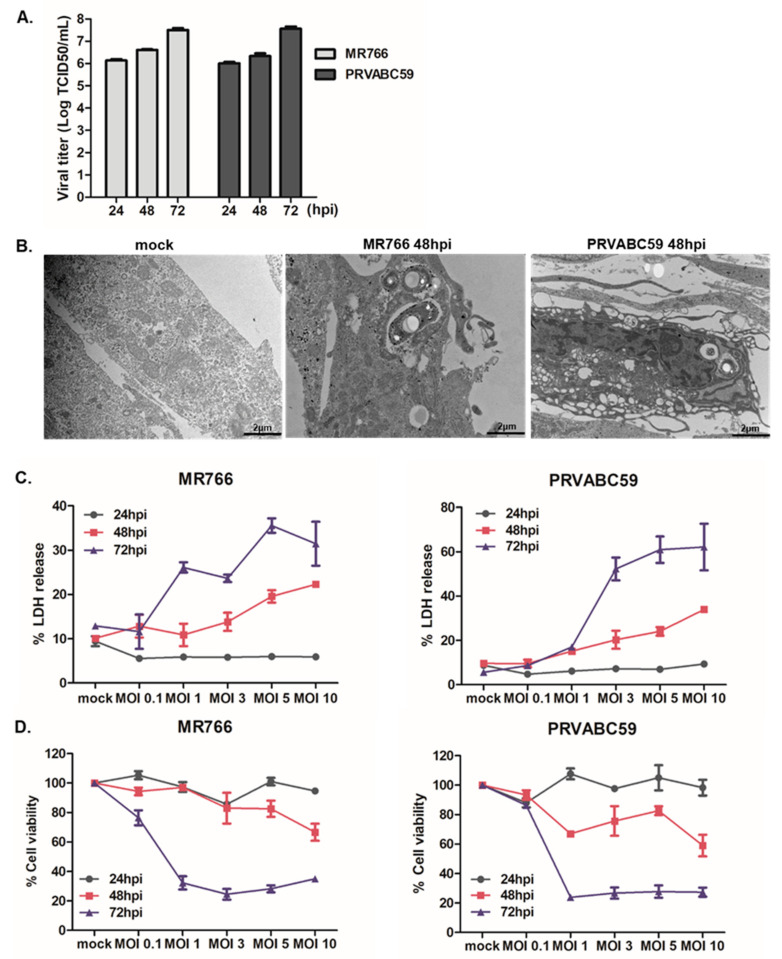
Cell viability and death during Zika virus (ZIKV) infection of hNPCs. (**A**) Viral titers were measured by TCID50 assay. Supernatant was collected from hNPCs infected with ZIKV (MR766 or PRVABC59) at MOI of 3 for various timepoints. TCID50 was calculated using the Spearman–Kärber method and expressed as TCID50/mL. Results represent means ± SD of two independent experiments. (**B**) hNPCs were infected with ZIKV (MR766 and PRVABC59) at MOI of 3. Transmission electron microscopy shows the presence of apoptotic cell death. (**C**) % LDH release was measured in cell supernatant of mock-infected cells vs. ZIKV-infected cells. Values were normalized to mock-infected cells. (**D**) Cell viability was measured at 24, 48, and 72 hpi by CellTiter Glo luminescence assay and normalized to mock-infected cells. Values represent the mean and standard deviation of three independent experiments.

**Figure 2:**
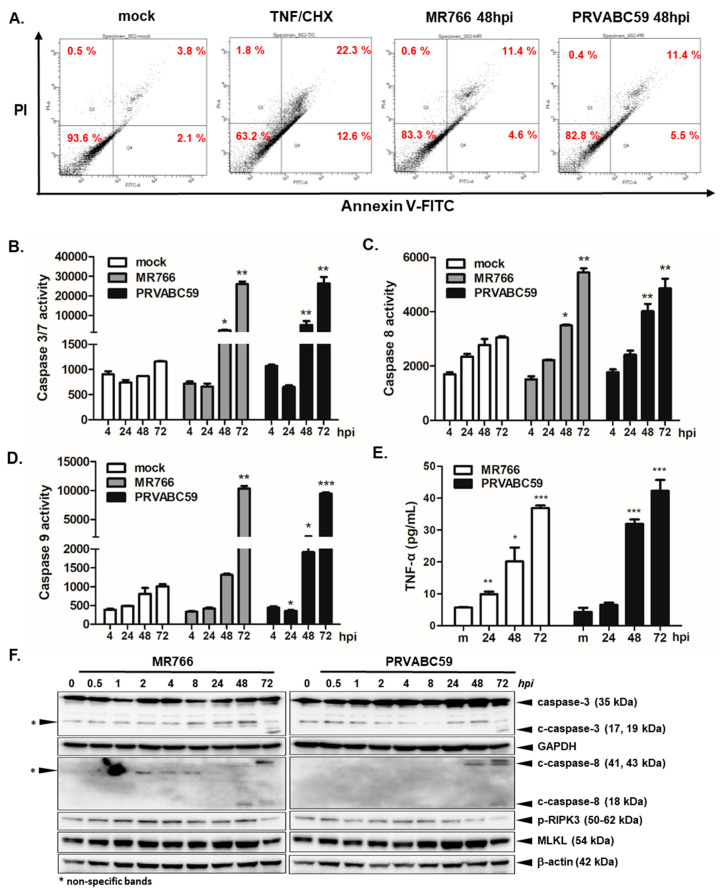
Caspase activity during Zika virus (ZIKV) infection of hNPCs. hNPCs were either stimulated with TNF-α and cycloheximide (TNF/CHX) or infected with mock or ZIKV at MOI of 3. (**A**) Flow cytometry analysis by AnnexinV5-FITC/PI staining shows % cells undergoing apoptosis. Activity of caspases 3/7 (**B**), 8 (**C**), and 9 (**D**) in mock- and ZIKV-infected cultures was determined using caspase luminescence assays after 24, 48, and 72 hpi. Data represented as mean ± SD. (* *p* < 0.05; ** *p* < 0.01; *** *p* < 0.001, compared with mock control). (**E**) TNF-α secretion level from ZIKV-infected hNPCs was measured by ELISA assay. * *p* < 0.05; ** *p* < 0.01; *** *p* < 0.001 versus mock-infected control cells. (**F**) hNPCs were infected with ZIKV at an MOI of 3 for the indicated times. The cleavage of caspase 3 and caspase-8, phosphorylated RIPK3, and MLKL protein level is shown by western blot analysis. The images are representatives of three independent experiments.

The cytopathic effects of ZIKV infection in hNPCs have been reported by several groups [6,7,8,9,12]. We first evaluated the infectivity of ZIKV in hNPCs. Cells were infected with ZIKV (MR766 or PRVABC59 strain) at MOI of 3, and ZIKV titers were measured by TCID50 assay. Similar to previous findings, hNPCs were highly susceptible to both MR766 and PRVABC59 ZIKV infection (Figure 1A). Transmission electron microscopy showed condensed fragmented nuclei and numerous autophagic vacuoles in the cytoplasm following ZIKV infection (Figure 1B). Next, we evaluated ZIKV-mediated cytotoxicity by measuring LDH release in cellular supernatant. Minimal cell death was observed at 24 h post infection (hpi), but there was an MOI-dependent increase in cell death starting at 48 hpi (Figure 1C). When cell viability was compared between MR766 and PRVABC59, there was a marked reduction in the number of viable cells following ZIKV infection, in comparison to mock-infected cells (Figure 1D). On the other hand, Annexin V5-FITC/PI staining revealed similar % cells going through apoptosis between MR766 and PRVABC59-infected hNPCs at 48 hpi (Figure 2A).

In order to determine the caspase activities of ZIKV-infected hNPCs, we next measured caspase activities by caspase luminescence assay at different timepoints. During early infection, no significant changes in caspase activities were observed. However, significantly increased levels of caspase 3/7, 8, and 9 activity were observed at 48 and 72 hpi, in accordance with the levels of cell viability and cell death shown in Figure 1 (Figure 2B–D). We also investigated differential secretion of inflammatory cytokines, such as TNF-α, following ZIKV infection as a possible mechanism of ZIKV-induced cell death. Interestingly, the TNF-α secretion level also increased in a time-dependent manner following ZIKV infection (Figure 2E). In accordance with the caspase activity assay, western blot confirmed upregulation of cleaved caspase 3 and caspase 8 expression levels starting at 48 hpi (Figure 2F). In order to examine additional cell death mechanisms, the expression levels of factors associated with necroptosis were examined by western blot. Surprisingly, there was an upregulation of mixed lineage kinase domain-like protein (MLKL), an essential regulator of necroptosis, along with receptor interacting protein kinase 3 (RIPK3), which recruits and phosphorylates MLKL.

### 3.2. ZIKV-Mediated Cell Death Is Involved in the Regulation of the Inflammatory Response

**Figure 3:**
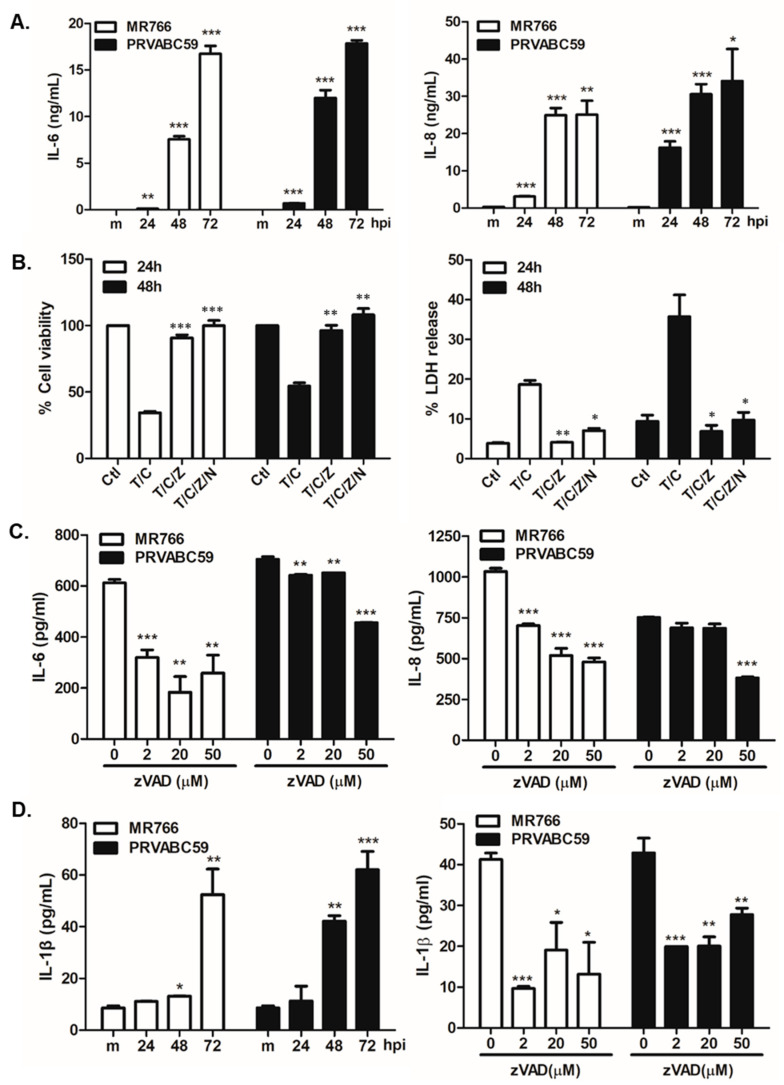
The effect of apoptosis inhibitors on inflammation during Zika virus (ZIKV) infection of hNPCs. (**A**) hNPCs were infected with ZIKV at MOI of 3. IL-6 and IL-8 secretion levels at 24, 48, and 72 hpi were measured by ELISA. (**B**) hNPCs were treated with TNF-α and cycloheximide (T/C), TNF-α, cycloheximide, and zVAD-fmk (T/C/Z), or TNF-α, cycloheximide, z-VAD-fmk, and Nec-1 (T/C/Z/N). % cell survival was determined by the CellTiter-Glo assay (**left**). % LDH release was measured in cell supernatants and the graph represents the mean and standard deviation of three independent experiments (**right**). * *p* < 0.05; ** *p* < 0.01; *** *p* < 0.001, compared to T/C-treated cells. (**C**) hNPCs were treated with 0, 2, 20, and 50 µM of z-VAD-fmk overnight and infected with ZIKV for 24 hpi. IL-6 and IL-8 secretion levels were determined by ELISA. * *p* < 0.05; ** *p* < 0.01; *** *p* < 0.001, compared to DMSO control-treated cells. (**D**) IL-1β secretion level was measured at 24, 48, and 72 hpi by ELISA (**left**). hNPCs were treated with 0, 2, 20, and 50 µM of z-VAD-fmk overnight and infected with ZIKV for 48 hpi. IL-1β secretion levels were determined by ELISA (**right**). * *p* < 0.05; ** *p* < 0.01; *** *p* < 0.001, compared to DMSO control-treated cells.

Previously, McGrath et al. reported a global gene expression analysis of ZIKV-infected hNPCs, including modulated expression levels of interferon response and inflammation/immunity-related genes [30]. Thus, we investigated whether ZIKV-induced apoptosis involves the production of inflammatory cytokines and chemokines. First, we examined the expression pattern of IL-6 and IL-8 during ZIKV infection. hNPCs were infected with MR766 or PRVABC59, and cell supernatant was collected at 24, 48, and 72 hpi. Protein expression levels of both IL-6 and IL-8 were upregulated at 24 hpi, and significantly elevated at 48 and 72 hpi (Figure 3A). The increased levels of IL-6 and IL-8 correlated with the amount of cell death observed in Figure 1.

Similar to apoptosis, necroptosis has recently been implicated as a host defense mechanism against various pathogens [31]. Components of both apoptosis and necroptosis can regulate each other’s activities, providing evidence for a mutual relationship between both cell death modalities. Therefore, to investigate the effect of blocking apoptosis or necroptosis on ZIKV-induced inflammation, we treated cells with drugs specifically known to inhibit apoptosis or necroptosis. Cells were treated with the following combinations: TNF-α and cycloheximide (T/C), TNF-α, cycloheximide, and z-VAD-fmk (T/C/Z), or TNF-α, cycloheximide, z-VAD-fmk, and necrostatin-1 (T/C/Z/N). z-VAD-fmk is a pan-caspase inhibitor that blocks apoptosis, and necrostatin-1 is a necroptosis inhibitor. As shown in Figure 3B, we observed that cell viability was greatly diminished upon T/C treatment, and z-VAD-fmk treatment was able to recover cell viability up to 90%. Given that z-VAD-fmk significantly recovered cell viability, the addition of Nec-1 did not cause a major difference in cell viability. This finding emphasizes the importance of apoptosis, rather than necroptosis, in ZIKV-mediated cell death (Figure 3B). Similar results were obtained from the LDH assay.

To test whether z-VAD-fmk can further modulate ZIKV-induced inflammatory cytokine production, hNPCs were treated with different doses of z-VAD-fmk, and infected with ZIKV for 24 h. IL-6 and IL-8 protein secretion levels were significantly reduced in z-VAD-fmk-treated samples, and these results show that apoptotic pathways are involved in the regulation of inflammation (Figure 3C). Given that previous literature suggests a role of inflammasome during ZIKV infection, we also evaluated the secretion levels of IL-1β, a key cytokine of inflammasome activation. Figure 3D shows that ZIKV infection in hNPCs led to an increased IL-1β secretion level and the inhibition of apoptosis with z-VAD-fmk resulted in the suppression of IL-1β, suggesting the important role of apoptosis signaling for IL-1β secretion.

### 3.3. TRAIL-Mediated Apoptosis Is Important for Controlling ZIKV-Mediated Cell Death in hNPCs

**Figure 4:**
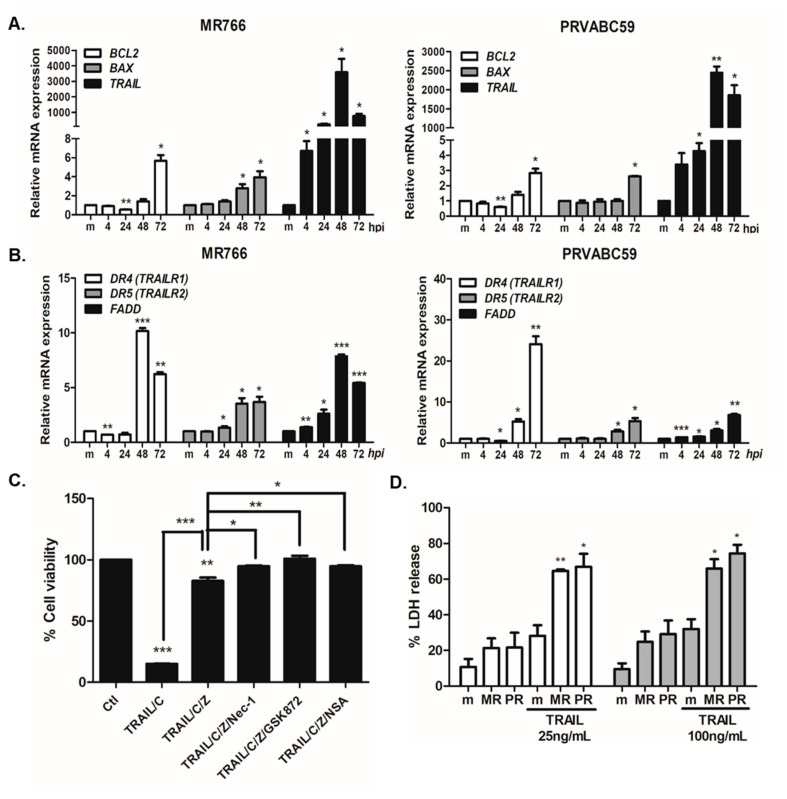
ZIKV infection results in increased expression of genes involved in TRAIL signaling, and TRAIL-mediated apoptosis augments ZIKV-induced cell death. (**A**,**B**) hNPCs were infected with ZIKV (MOI of 3) and RNA was collected at 4, 24, 48, and 72 hpi. mRNA levels of *BCL2*, *BAX*, *TRAIL*, *TRAILR1*, *TRAILR2*, and *FADD* were measured over time using qRT-PCR. The expression of genes was normalized to β-actin and the levels of expression for the control mock-infected group were arbitrarily set to 1. * *p* < 0.05; ** *p* < 0.01; *** *p* < 0.001, compared with mock-infected cells. (**C**) hNPCs were treated with TRAIL and cycloheximide (TRAIL/C), TRAIL, cycloheximide, and z-VAD-fmk (TRAIL/C/Z), or TRAIL, cycloheximide, z-VAD-fmk, and Nec-1 (TRAIL/C/Z/N), TRAIL, cycloheximide, zVAD, and GSK872 (TRAIL/C/Z/N), TRAIL, cycloheximide, z-VAD-fmk, and NSA (TRAIL/C/Z/NSA). % cell survival was determined by CellTiter-Glo assay. (**D**) % LDH release was measured in the supernatants of mock- or ZIKV- infected cells followed by recombinant TRAIL treatment (25 or 100 ng/mL). * *p* < 0.05; ** *p* < 0.01; *** *p* < 0.001, compared with ZIKV-infected DMSO-treated cells.

Next, we examined apoptosis-related gene expression profiles of MR766- vs. PRVABC59-infected hNPCs. BCL2 family members form hetero- or homodimers and act as pro- or anti-apoptotic regulators that are involved in a wide variety of cellular activities [32]. BAX is a member of the Bcl-2 gene family that heterodimerizes with BCL2, and functions as an apoptotic activator. TRAIL is a member of TNF family, which triggers apoptosis pathways via binding to and activating death receptors TRAILR2 (DR4) and TRAILR1 (DR5), subsequently recruiting Fas-associated death domain (FADD), which drives the recruitment of pro-caspase 8 into the death receptor complex and initiates apoptosis pathways [33,34]. Among apoptosis-related genes, we detected the *TRAIL* gene to be highly upregulated upon both MR766 and PRVABC59 infection (Figure 4A). Furthermore, qRT-PCR results revealed a significant increase in *TRAILR2 (DR4)* and *TRAILR1 (DR5)* expression in both MR766-and PRVABC59-infected cells at 48 and 72 hpi. Transcript levels of *TRAILR2 (DR4)* and adaptor protein *FADD* were also enhanced upon ZIKV infection (Figure 4B).

To test whether hNPCs are sensitive to TRAIL-mediated apoptosis, we treated hNPCs with TRAIL in combination with apoptosis or necroptosis inhibitors. Figure 4C indicates that TRAIL caused more than 80% cell death in hNPCs, and z-VAD-fmk treatment greatly recovered the cell viability. Interestingly, cells treated with TRAIL/C/Z combined with necroptosis inhibitors, such as Nec-1, GSK872, or NSA, resulted in higher cell viability in comparison to a single treatment of z-VAD-fmk (Figure 4C). In addition, we evaluated whether TRAIL-mediated signaling can modulate ZIKV-mediated cell death. TRAIL treatment in ZIKV-infected cells led to a significant increase in % cytotoxicity, as compared with ZIKV-infected cells alone, suggesting the importance of TRAIL-mediated apoptosis during ZIKV infection (Figure 4D).

**Figure 5:**
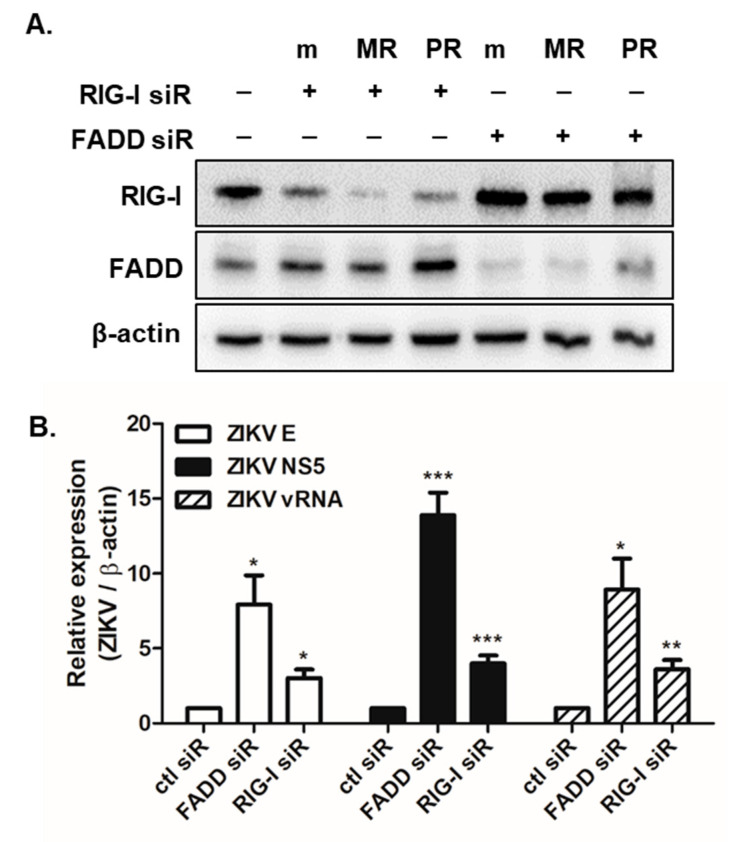
The knockdown of TRAIL adaptor FADD results in increased ZIKV replication. (**A**) A549 cells were transfected with either control, RIG-I, or FADD siRNA. After 24 h, cells were mock (m)- or MR766 (MR)- or PRVABC59 (PR)-infected for 24 h. The knockdown efficiency of RIG-I or FADD siRNA was determined by measuring the expression levels of RIG-I or FADD protein by western blot. Data representative of three independent experiments are shown. (**B**) mRNA levels of ZIKV E, NS5, and vRNA were measured using qRT-PCR. Gene expression was normalized to β-actin and the expression level of control siRNA-transfected cells infected with ZIKV was arbitrarily set to 1. * *p* < 0.05; ** *p* < 0.01; *** *p* < 0.001 versus control siRNA-transfected ZIKV-infected cells.

For further evaluation of the effects of TRAIL signaling on viral replication efficiency, FADD siRNA was transfected into A549 cells. siRNA-induced downregulation of FADD expression was confirmed by Western blot analysis (Figure 5A). FADD siRNA transfection resulted in significantly elevated gene expression of ZIKV NS5, E, and vRNA, suggesting that TRAIL signaling negatively regulates viral replication (Figure 5B).

## 4. Discussion

Multiple studies of cell death mechanisms in ZIKV-infected cells demonstrate that ZIKV infection can have variable cytotoxic effects depending on the viral strain and the cell type [9,10,11,12,35,36,37]. In particular, a recent study by Turpin et al. found that although there was no significant detection of apoptosis at 24 hpi, a significant increase in ZIKV-mediated apoptosis was detected at 48 and 72 hpi in A549 cells [37]. Similar to previously reported findings, our data also confirm that both MR766 and PRVABC59 are able to directly infect hNPCs and induce similar levels of cytotoxic effects. Furthermore, our TCID50 assay revealed that viral production levels of MR766 and PRVABC59 were similar, a finding that is in line with the study by Ferraris et al. [14]. Interestingly, differential cytotoxicity of the ZIKV strains was indicated by several groups [7,30,38,39,40]. Devhare et al. observed that MR766 infection induced phosphorylation of H2AX along with PARP and caspase-3 cleavage [9]. On the other hand, PRVABC59 infection in hNPCs resulted in p53 phosphorylation and induction of p21 and PUMA, all of which are factors indicative of cell cycle arrest. Our data, however, did not reveal a clear difference in the cytotoxic levels between MR766- and PRVABC59-infected cells. The discrepancy may be due to use of different sources and strains of ZIKV used in our study, and the comparable rates of virus production likely resulted in similar cytotoxic effects for the two ZIKV strains.

Reports have shown that ZIKV-induced cytopathic effects occur due to the activation of various cell death pathways in the host cell [9,11,12,17,35,36]. For example, ZIKV-induced apoptosis of neural cells has been addressed by several studies. Souza et al. attributes the massive death of neural stem cells following ZIKV infection to mitotic dysfunctions, including the presence of extra centrosomes and abnormal chromosomes [12]. Slomnicki et al., on the other hand, emphasizes the presence of ribosomal stress like nucleolar disruption as the mediator of apoptosis of ZIKV-infected neural cells [11]. Both studies show that ZIKV-induced neuronal apoptosis likely plays a significant role in the neurodevelopmental defects that are observed following congenital ZIKV infection.

Apoptosis is not the only cell death pathway that ZIKV regulates. According to Daniels et al., neuronal ZIKV infection activated ZBP1 and RIPK1/3 pathways, which are essential components of the virus-induced necroptotic signaling pathway [17]. RIPK1/3 orchestrates a type of cell death known as necroptosis via activation of the executioner called mixed lineage kinase domain-like protein (MLKL), and necroptosis is associated with promoting pathogen clearance and host cell defense. In line with this recent finding, we wanted to investigate whether RIPK signaling modulates ZIKV-mediated cell death, given that ZIKV infection of hNPCs led to highly upregulated production of inflammatory cytokines and chemokines. As shown in Figure 3B, cell viability was greatly reduced upon T/C treatment, and this effect was alleviated up to 90% by z-VAD-fmk, showing that apoptosis pathway is a major cell death pathway for ZIKV-mediated response, although elevated levels of phosphorylated RIPK3 and MLKL protein expression at 48 and 72 hpi were also observed by Western blot (Figure 2F). These findings suggest that various mechanisms of cell death are associated with ZIKV infection in hNPCs.

Massive vacuolization is another ZIKV-induced cytopathic effect that is similar to the cytoplasmic vacuoles that form during paraptosis, which is a caspase-independent non-apoptotic cell death. According to Monel et al., the ZIKV-induced vacuoles in dermal fibroblasts, astrocytes, and HeLa cells originate from the endoplasmic reticulum, which is a common site for the replication of flaviviruses and cell death [10,41,42,43]. Our TEM analysis also revealed the presence of numerous small autophagic vacuoles in the cytoplasm along with condensed fragmented nuclei (Figure 2B). Previously, Liang et al. showed that ZIKV proteins NS4 can activate autophagic signaling via abrogating Akt-mTOR pathways in hNPCs [44] and our unpublished data also indicate that ZIKV NSs are involved in the modulation of mitophagy. Therefore, it will be interesting to further characterize the link between autophagy/mitophagy and cell death for the contribution of severe complications in neonatal brains.

Host–pathogen interactions are important determinants of virus-induced cytopathic effects, such as the previously mentioned forms of cell death, and TRAIL has been characterized as a key mediator of such interactions during immune responses to viral infections. Fas-associated protein with death domain (FADD) is an essential adaptor for death receptor-mediated apoptosis. Our data with FADD knockdown cells show that FADD is important for regulation of ZIKV-mediated cell death mechanisms. Recent studies demonstrate the dual function of TRAIL within the immune system, where TRAIL signaling may lead to pathogen persistence and immune suppression or pathogen clearance via cell death [19,20,21,22,45]. Binding of TRAIL to specific death receptors can result in activation of apoptosis and necroptosis of virus-infected cells as part of the host defense mechanism [46,47]. Therefore, the upregulation of TRAIL expression, as shown in Figure 4, may be part of the antiviral response mounted by ZIKV-infected hNPCs. However, viruses have also been shown to inhibit cell death pathways in order to promote viral replication and persistence, as well as using TRAIL-mediated mechanisms to induce cell death of non-infected cells [48]. The multifaceted characteristics of TRAIL have been well established by previous studies, and the ability of TRAIL to mediate protective or pathogenic mechanisms depends on the cell types and the expression of death receptors. Our findings provide insights into the involvement of TRAIL in the morphological and cellular changes induced by ZIKV in hNPCs and emphasize the need for further studies to identify the function of TRAIL-mediated signaling in other ZIKV-susceptible cells.

Inflammation represents an additional mechanism of host responses to pathogens, and ZIKV infection in newborns has been shown to induce the production of inflammatory cytokines [49,50]. NOD-like receptors (NLRs) are responsible for the assembly of inflammasomes, which are signaling complexes involved in caspase-1-dependent innate immunity involving cytokines, such as IL-1β, and in the induction of cell death by pyroptosis [16,51,52]. ZIKV-induced microcephaly has highlighted monocytes as potential carriers that can cross the placenta to transmit ZIKV to the fetus. Khaiboullina et al. and Wang et al. demonstrated that ZIKV infection in monocyte-derived cells was associated with prolonged activation of NLRP3 inflammasome, as shown by the increased levels of IL-1β and caspase-1 secretion, and Zheng et al. associated ZIKV-induced inflammasome activation with the evasion of type I IFN-mediated host immune response [53,54,55]. Figueiredo et al. also detected the expression of TNF-α, in addition to IL-1β, in ZIKV-infected brains of mice, while investigation of fatal ZIKV cases in human patients by de Sousa et al. revealed inflammasome activation in the neural parenchyma, as evident from the upregulation of NLRP3 and caspase 1 expression levels [56,57]. Our data demonstrating ZIKV-induced IL-1β secretion from hNPCs (Figure 3D), as well as previous findings on ZIKV-induced inflammasome, highlight the potential role of ZIKV-mediated inflammasome activation in neurodevelopmental defects, including microcephaly.

In conclusion, our study demonstrates that ZIKV triggers changes in the expression of genes involved in apoptosis and leads to activation of the cytopathic effect via the TRAIL-mediated apoptosis pathway. Accompanied by our study, multiple reports have shown that morphological cellular changes induced by ZIKV are capable of modulating various forms of cell death. Therefore, the cytopathic effects observed during ZIKV infection of neural cells, in particular, deserve further attention in order to characterize the mechanisms underlying the serious congenital neurodevelopmental defects caused by fetal ZIKV infections.

## Figures and Tables

**Table 1:** Primer sequences used in this study.

Gene List	Primer-Forward	Primer-Reverse
*BCL2*	CTGCACCTGACGCCCTTCACC	CACATGACCCCACCGAACTCAAAGA
*BAX*	CCCGAGAGGTCTTTTTCCGAG	CCAGCCCATGATGGTTCTGAT
*FADD*	GCTGGCTCGTCAGCTCAAA	ACTGTTGCGTTCTCCTTCTCT
*TRAIL*	TGCGTGCTGATCGTGATCTTC	GCTCGTTGGTAAAGTACACGTA
*TRAIL-R1*	ACCTTCAAGTTTGTCGTCGTC	CCAAAGGGCTATGTTCCCATT
*TRAIL-R2*	GCCCCACAACAAAAGAGGTC	AGGTCATTCCAGTGAGTGCTA
*ZIKVvRNA*	AGATGACTGCGTTGTGAAGC	GAGCAGAACGGGACTTCTTC
*ZIKV E*	TATCAGTGCATGGCTCCCAGCATA	TCCTAAGCTTCCAAAGCCTCCCAA
*ZIKV NS5*	CCTTGGATTCTTGAACGAGGA	AGAGCTTCATTCTCCAGATCAA
*β-actin*	GAGCACAGAGCCTCGCCTTT	ACATGCCGGAGCCGTTGTC
